# Effects of dietary nitrate supplementation on peak oxygen uptake following 10 days of Ramadan fasting in healthy adults

**DOI:** 10.1113/EP093800

**Published:** 2026-08-18

**Authors:** Mikail M. Bhimani, Julian C. Bommarito, Natalie I. Miners, Pardeep K. Khangura, Rileigh K. Stapleton, Ashly Sharma, Johan S. Thiessen, Nicolas O. Cyr, Devin G. McCarthy, Jianzhang Dong, Clara E. Cho, Philip J. Millar

**Affiliations:** ^1^ Human Cardiovascular Physiology Laboratory, Department of Human Health Sciences University of Guelph Guelph Ontario Canada; ^2^ Department of Kinesiology University of Guelph‐Humber Etobicoke Ontario Canada; ^3^ Department of Human Health Sciences University of Guelph Guelph Ontario Canada

**Keywords:** dietary nitrate, oxygen uptake, ramadan

## Abstract

Ramadan is a sacred religious practice observed by Muslims that involves abstaining from food and water from sunrise to sunset for 1 month. Prior work has reported that peak oxygen uptake (${\dot{V}}_{O_{2}\mathrm{peak}}$) can decline during Ramadan in parallel with reduced physical activity. We investigated whether dietary nitrate supplementation could attenuate the decrease in ${\dot{V}}_{O_{2}\mathrm{peak}}$ during 10 days of Ramadan fasting. In a randomized design, 21 healthy participants (13 females) were assigned to ingest 70 mL of beetroot juice (∼6.45 mmol nitrate; *n *= 10), twice a day for 10 days consecutively, or a non‐supplement control (*n *= 11). The ${\dot{V}}_{O_{2}\mathrm{peak}}$ was assessed by indirect calorimetry during a maximal cycle ergometer ramp test. After 10 days of Ramadan fasting, the change in body mass was not different between groups [change (∆), −0.91 ± 1.3 vs. −0.27 ± 1.2 kg (control vs. nitrate), *P *= 0.25], nor were the changes in resting systolic (*P *= 0.21) or diastolic (*P *= 0.53) blood pressure or urine specific gravity (*P *= 0.17). Subjective sleep quality declined over time in both groups (*P *< 0.01). Participants self‐reported a decline (*n *= 17) or no change (*n *= 4) in regular physical activity, and the number of exercise bouts during the 10 days of Ramadan did not differ between groups (2.5 ± 2.0 vs. 3.3 ± 2.4, *P *= 0.44). Absolute ${\dot{V}}_{O_{2}\mathrm{peak}}$ decreased in the control group compared with the dietary nitrate supplementation group (∆, −155 ± 168 vs. +67 ± 162 mL min^−1^, *P *< 0.01), as did ${\dot{V}}_{O_{2}\mathrm{peak}}$ relative to body mass (∆, −2.4 ± 2.5 vs. +0.3 ± 1.9 mL kg^−1^ min^−1^, *P *= 0.02) and peak power output (∆, −12 ± 16 vs. ∆+2 ± 10 W, *P *= 0.04). Dietary nitrate supplementation might represent a practical nutritional strategy to preserve aerobic capacity during Ramadan fasting.

## Abstract (highlights)

- **What is the central question of this study?**
  Does dietary nitrate supplementation attenuate the reduction in peak oxygen uptake during 10 days of Ramadan fasting?
- **What is the main finding and its importance?**
  Daily dietary nitrate supplementation using beetroot juice mitigated the decline in peak oxygen uptake observed during 10 days of Ramadan fasting, independent of changes in body mass, hydration status and sleep quality. These findings highlight that dietary nitrate supplementation might represent an accessible nutritional aid to help preserve cardiorespiratory capacity during periods of reduced physical activity and/or behavioural changes in dietary intake and sleep quality.

## 1 INTRODUCTION

Each year, Muslims participate in Ramadan, a month of intermittent daily fasting undertaken for spiritual reflection and self‐discipline (Hillier et al., 2024). Those who practice Ramadan abstain from food and water from sunrise to sunset for ∼29–30 days, with exemptions granted for those who are elderly, sick, pregnant or menstruating (Hillier et al., 2024). This prolonged period of daytime fasting induces measurable physiological changes, including shifts in energy metabolism (i.e., reductions in fasting glucose and insulin concentrations) (Gnanou et al., 2015; Sweileh et al., 1992), a reduction in fat‐free mass (Fernando et al., 2019; Najafi et al., 2023), trainsient hypohydration (Mustafa et al., 1978; Shirreffs, 2003; Sweileh et al., 1992) and reductions in resting blood pressure (Al‐Jafar et al., 2021). In addition, Ramadan is associated with several behavioural adaptations, such as reduced sleep duration (Roky et al., 2001) and lower habitual physical activity (Farooq et al., 2021; Jandali et al., 2024; Lipert et al., 2021). Collectively, these changes might influence physiological responses to exercise, with practical implications for athletes, shift‐workers and individuals engaged in physically demanding occupations.

Several studies have examined the effects of Ramadan fasting on peak oxygen uptake rate (${\dot{V}}_{O_{2}\mathrm{peak}}$). Roy and Bandyopadhyay (2015) reported a progressive decline in ${\dot{V}}_{O_{2}\mathrm{peak}}$ across Ramadan, with values returning to baseline within 2 weeks of fasting cessation (Roy & Bandyopadhyay, 2015). In contrast, other work has observed reductions in ${\dot{V}}_{O_{2}\mathrm{peak}}$ only during the first 1–2 weeks of Ramadan (Sweileh et al., 1990) or reported that ${\dot{V}}_{O_{2}\mathrm{peak}}$ does not change with Ramadan (Brisswalter et al., 2011). Collectively, these findings may suggest a drop in maximal aerobic capacity during the early stages of Ramadan, with mixed results towards the end of the month. The mechanisms underpinning these discrepant between‐study responses remain unclear. During the early phase of Ramadan, reduced fluid intake (Sweileh et al., 1990) could decrease plasma volume (Bouhlel et al., 2013), potentially constraining maximal cardiac output and limiting ${\dot{V}}_{O_{2}\mathrm{peak}}$. Concurrently, the beginning of Ramadan is associated with a rapid decline in regular physical activity (Farooq et al., 2021), which could result in skeletal muscle atrophy, and a decline in sleep quality, which might be linked to impaired exercise performance (Lipert et al., 2021). Notably, other forms of intermittent fasting have been associated with maintained or even improved ${\dot{V}}_{O_{2}\mathrm{peak}}$ (Correia et al., 2020), suggesting that factors unique to Ramadan, such as daytime fluid restriction and altered sleep–wake cycles, might contribute to potential reductions in aerobic capacity. To date, no intervention strategies have been evaluated to attenuate possible declines in ${\dot{V}}_{O_{2}\mathrm{peak}}$ during Ramadan fasting.

Dietary nitrate supplementation has emerged as a potential nutritional strategy to improve exercise performance (Poon et al., 2025). Commonly ingested in the form of beetroot juice, dietary nitrate can increase ${\dot{V}}_{O_{2}\mathrm{peak}}$ in both healthy (Vanhatalo et al., 2010) and clinical (Coggan et al., 2018) populations. Dietary nitrate can also reduce blood pressure at rest (Norouzzadeh et al., 2024) and during exercise (Benjamim et al., 2024). After ingestion, nitrate is reduced to nitrite by facultative anaerobic oral bacteria and converted further, to nitric oxide, via endogenous nitrite reductases (Lundberg et al., 2008). Nitric oxide is a potent vasodilator that can enhance skeletal muscle perfusion and oxygen delivery and might improve matching of oxygen supply to metabolic demand (Jones, 2014). In addition to its vascular effects, dietary nitrate supplementation can reduce the oxygen cost of submaximal exercise (Gao et al., 2021), increase maximal muscle power (Coggan et al., 2021) and improve contractile efficiency by reducing the ATP cost of force production (Bailey et al., 2010; Fulford et al., 2013). Other studies have demonstrated that dietary nitrate can attenuate reductions in ${\dot{V}}_{O_{2}\mathrm{peak}}$ following blood donation (McDonagh et al., 2016) or normobaric hypoxia (Cocksedge et al., 2020). Whether dietary nitrate supplementation can attenuate the decline in ${\dot{V}}_{O_{2}\mathrm{peak}}$ during Ramadan fasting has not been explored.

Therefore, the aim of the present study was to investigate the effects of daily dietary nitrate supplementation on ${\dot{V}}_{O_{2}\mathrm{peak}}$ following 10 days of Ramadan fasting in healthy adults. We hypothesised that dietary nitrate supplementation would mitigate declines in ${\dot{V}}_{O_{2}\mathrm{peak}}$ in comparison to the control group. Secondary variables included examining the changes in body mass, hydration status and sleep quality.

## 2 MATERIALS AND METHODS

### 2.1 Participants

Participants aged 18–31 years old were recruited and screened using a general health questionnaire. All participants self‐reported to be free of any chronic disease or acute health conditions and were not taking any prescription medications. Participants reported no prior use of antibacterial mouthwash over the past 30 days and were reminded to abstain throughout the study, because elimination of oral bacteria can reduce the conversion of dietary nitrate to nitrite. All participants were recreationally active, with the exception of one non‐endurance athlete (University of Guelph wrestling team). All procedures were approved by the University of Guelph Research Ethics Board in accordance with the *Declaration of Helsinki* (REB#1444‐1). Each participant was required to provide informed written consent prior to testing. This study was registered on ClinicalTrials.gov (NCT06767176).

### 2.2 Study design and protocol

The study was a randomized controlled trial with a pre–post design. Participants were block randomized, stratified for sex (randomizer.org), into either the control or dietary nitrate group. Participants in the dietary nitrate group were instructed to consume two 70 mL shots of commercially available beetroot juice (Beet‐It Sport, James White Drinks Ltd, Belfast, UK) per day, equating to ∼800 mg or ∼12.9 mmol of nitrate. One dose of beetroot juice was consumed upon waking, prior to the pre‐fasting meal, and the second was consumed prior to the first evening meal. Participants in the control group did not receive any supplementation. Participants were not informed of the study hypothesis at any point in the trial, in an attempt to minimize expectancy effects.

Each participant was required to visit the laboratory for testing on two separate occasions (before Ramadan and after ≥10 consecutive days of Ramadan fasting). The majority of participants (*n *= 16) fasted from the first day of Ramadan (1 March 2025) until the 10th or 11th day (10/11 March 2025). Based on the location (Guelph, Ontario, Canada) and time of year, fasting lasted for ∼12–13 h each day. Owing to the number of participants, post‐testing was spread across 2 days. Participants in the dietary nitrate group who were scheduled to return on 10 March were instructed to begin supplementation on the night of 28 February, and those scheduled for 11 March were instructed to start beetroot juice supplementation with their pre‐fasting meal on 1 March. This allowed for all participants to have one remaining beetroot juice supplementation during the pre‐fast meal of their post visit. Five female participants were scheduled to commence their menstrual cycle during the above dates, permitting them to stop fasting (Hillier et al., 2024). To accommodate this, these participants waited to start their 10 day fasting window until after their menstrual period and returned to the laboratory 10 consecutive days later for the second study visit. For transparency, one female each completed the second visit on the 12th, 19th and 24th day of Ramadan, and two females completed the second visit on the 20th day of Ramadan. All study testing was completed between 24 February 2025 and 24 March 2025.

Prior to each study visit, participants were instructed to abstain from alcohol, recreational drugs, caffeine and strenuous exercise for 24 h. The majority of participants (*n *= 17) completed the second visit at the same time of day as the first visit (±2 h). At the commencement of each study visit, participants were asked to provide a urine sample to enable the measurement of urine specific gravity using a digital hand‐held pen refractometer (ATAGO USA, Bellevue, WA, USA) to estimate hydration status. Next, the participant was taken to a private area, where they sat quietly, with their back and arms supported, feet flat on the floor and legs uncrossed, for 5 min, after which six discrete measurements of brachial blood pressure and heart rate were taken at 1 min intervals (BPTru Medical Devices, Coquitlam, BC, Canada). The average of the last five measurements was calculated and used as resting blood pressure and heart rate. In 15 participants, venous blood was sampled from an antecubital vein near the cubital fossa, collected into potassium EDTA vacutainers and immediately put on ice. Blood samples were centrifuged within 90 min at 1000*g* for 10 min at 4°C. Plasma was removed and aliquoted into cryovials and stored at −80°C for later analysis. Next, anthropometric measurements were recorded, and participants were instrumented with six surface electrodes according to the manufacturer's specification to estimate cardiac output using thoracic impedance cardiography (Physioflow, Manatec Biomedical, France). This system has been validated for use in healthy individuals during maximal exercise testing (Richard et al., 2001). Our group recently reported greater measurement precision of peak cardiac output with impedance cardiography than with inert gas rebreathing using nitrous oxide (McCarthy et al., 2025). After a brief calibration period at rest, participants completed an incremental exercise test to maximal voluntary exertion using an electromagnetically braked cycle ergometer (Lode Excalibur Sport v.2.0, Groningen, The Netherlands). The protocol consisted of a 5 min warm‐up at 50 W, followed by a ramp increase of 1 W every 2 s (30 W min^−1^). Pedalling cadence was chosen by the participant and required to be between 70 and 90 rpm during the 50 W intensity and ≥60 rpm during the ramp protocol. Brachial artery blood pressure was monitored every 90 s during exercise using a motion‐tolerant, ECG‐gated, automated auscultatory device designed for exercise testing (Tango M2; SunTech Medical Inc., NC, USA). Ratings of perceived exertion were collected after each blood pressure measurement using a 6–20 Borg scale. Gas exchange and ventilatory variables were measured breath by breath using a metabolic cart (Quark CPET, COSMED, Rome, Italy). The exercise test concluded once participants were unable to maintain a cadence of 60 rpm or stopped cycling owing to volitional fatigue. A 5 min recovery cool‐down was performed at 0 W after the ramp protocol concluded. All exercise tests achieved a respiratory exchange ratio of >1.1 and a rating of perceived exertion of ≥16, which did not differ between study visits. To confirm that exercise tests reached maximal efforts, we used the criteria proposed by Wagner et al. (2020), calling for at least two of the following: (1) a respiratory exchange ratio > 1.13; (2) an age‐predicted maximal heart rate (210 − age)  ≥ 96%; and/or (3) an age‐predicted maximal heart rate (208 × 0.7 age)  ≥ 93%.

### 2.3 Plasma nitrate/nitrite

Plasma concentrations of nitrate and nitrite (NO_x_) were measured using a commercially available nitrate/nitrite colorimetric assay kit (Abnova, Taipei City, Taiwan; catalogue no. KA1345), containing predetermined standards and Griess Reagents. Plasma samples were deproteinized using ultrafiltration with 30 kDa spin filters, diluted 1:2 using NO_x_ assay buffer, then immediately assayed in duplicates according to the manufacturer's instructions. Absorbance values were averaged, and correction was performed by subtracting the absorbance of blank standard from each measurement. Total NO_x_ concentrations were calculated using the linear regression of: $\frac{\mathrm{corrected}\;\mathrm{absorbance}-(y-\mathrm{intercept})}{\mathrm{slope}}\times 2.5\times d\mathrm{ilution}\;\mathrm{factor}\;\mathrm{of}\;1:2$ based on the constructed standard curve. The intra‐assay coefficient of variation was 6.5%.

### 2.4 Sleep and behaviour tracking

Participants were asked to complete the Pittsburgh Sleep Quality Index questionnaire to track sleep quality changes during the Ramadan period. For the post‐fasting questionnaire, participants were specifically asked to comment on changes in sleep over the 10 day period rather than the usual 30 day period, as described in the original questionnaire. A Pittsburgh Sleep Quality Index score above five was considered indicative of poor sleep (Mollayeva et al., 2016). Three participants did not complete the pre‐Ramadan sleep questionnaire and were excluded from sleep analysis. During the second study visit, participants were also asked to complete a subjective questionnaire regarding physical activity patterns during the Ramadan period. This survey asked how many times they exercised during the 10 day Ramadan period and whether exercise frequency/intensity/duration changed.

### 2.5 Data analysis

This study was powered to detect a change in the primary variable, ${\dot{V}}_{O_{2}\mathrm{peak}}$. An a priori sample size calculation was computed using G*Power (v.3.1.9.7) based on past work showing a change in ${\dot{V}}_{O_{2}\mathrm{peak}}$ from 44 to 36 mL kg^−1^ min^−1^ during 16 days of Ramadan. Estimating a difference in the pre–post change (∆) in relative ${\dot{V}}_{O_{2}\mathrm{peak}}$ between the two independent groups of ∆ = −6 ± 4 mL kg^−1^ min^−1^, with an α of 0.05 and power of 0.80, we required an estimated nine participants per group. All data were collected blinded to group allocation. The data were filtered using a 30 s rolling average, and haemodynamic parameters obtained from cardiac impedance were averaged over 10 s epochs. Maximal heart rate and peak power output were defined as the highest values achieved during the ramp test. The rise in systolic blood pressure per metabolic equivalent was calculated as the change in systolic blood pressure divided by the maximal metabolic equivalent minus one (Hilton et al., 2024). Arteriovenous oxygen difference was calculated using the Fick principle, where oxygen uptake was divided by cardiac output (Basnet & Rout, 2026). The ${\dot{V}}_{O_{2}\mathrm{peak}}$ was defined as the highest value achieved after applying a 30 s rolling average to the raw data. The first ventilatory threshold and respiratory compensation point were identified using breath‐by‐breath metabolic data and analysed using a freely accessible online app (Keir et al., 2022). Exercise thresholds were assessed blinded by two examiners. In cases where thresholds were ≤100 mL between examiners, values were averaged, while thresholds of >100 mL required a third examiner, with the two closest values averaged.

### 2.6 Statistical analysis

Baseline characteristics were compared using unpaired Student's *t*‐tests. All pre–post data were analysed using linear mixed‐model analyses. Fixed effects included group, time, and group × time. To account for repeated measurements, random intercepts were included for participants. As an exploratory analysis, sex was also added as a fixed effect to examine the group × time × sex interaction. Bonferroni corrections were used when applicable. Effect sizes are reported as partial eta squared (η^2^_p_). For linear mixed models, η^2^_p_ was calculated from the *F*‐statistic and its associated numerator and denominator degrees of freedom. Statistical analyses were conducted using IBM SPSS (v.28, Armonk, NY, USA). A *P*‐value of <0.05 was considered statistically significant. All variables are reported as the mean ± SD unless otherwise noted.

## 3 RESULTS

Twenty‐four participants (15 female) were recruited. Three participants were excluded prior to analysis owing to an inability to complete the maximal exercise test (*n *= 1) or the presence of an initially undisclosed chronic health condition (*n *= 2). The final sample comprised 21 participants (control, *n *= 11; dietary nitrate, *n *= 10). Owing to technical issues with the metabolic cart during the post‐intervention visit of one participant, complete pre–post ${\dot{V}}_{O_{2}\mathrm{peak}}$ and peak power output data were available for 20 participants (11 control and 9 dietary nitrate). Cardiac output was collected in n = 18 (control, *n* = 11; dietary nitrate, *n* = 7). Exercise thresholds were calculated in n = 17 (control, *n* = 9; dietary nitrate, *n* = 8).

At baseline, participant characteristics (Table 1) were comparable between groups for height [167 ± 6 vs. 163 ± 9 cm (controls vs. nitrate); *P *= 0.33], body mass (67 ± 12 vs. 61 ± 16 kg; *P *= 0.35), resting heart rate (78 ± 12 vs. 73 ± 9 beats min^−1^; *P *= 0.27), systolic blood pressure (112 ± 11 vs. 102 ± 11 mmHg; *P *= 0.06), diastolic blood pressure (75 ± 8 vs. 70 ± 6 mmHg; *P *= 0.13), ${\dot{V}}_{O_{2}\mathrm{peak}}$ (33.8 ± 7.1 vs. 30.8 ± 8.3 mL kg^−1^ min^−1^; *P *= 0.38) and peak power output (220 ± 64 vs. 181 ± 71 W; *P *= 0.20). Age was statistically higher in the control versus nitrate group (22 ± 4 vs. 19 ± 1 years, *P *= 0.02). Most participants self‐reported a decline (*n *= 17) or no change (*n *= 4) in regular physical activity, but the number of exercise bouts during the 10 days of Ramadan did not differ between groups (2.5 ± 2.0 vs. 3.3 ± 2.4, *P *= 0.44).

Plasma NO_x_ levels increased in those undergoing dietary nitrate supplementation (∆, 36 ± 90 vs. 358 ± 132 µM, η^2^_p_ = 0.56, *P *< 0.001; Figure 1). A main effect of time was detected for sleep quality (η^2^_p_ = 0.48, *P *< 0.01), urine specific gravity (η^2^_p_ = 0.30, *P *= 0.01) and body mass (η^2^_p_ = 0.20, *P *= 0.04), but no group × time interactions were observed (all *P *> 0.17; Figure 2). The loss in body mass was unrelated to changes in hydration status (*r* = −0.22, *P *= 0.32).

**FIGURE 1:**
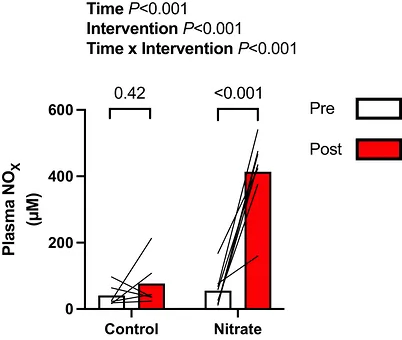
Changes in plasma concentrations of nitrate and nitrite (NO_x_) following the 10 day Ramadan fasting intervention with or without dietary nitrate supplementation. Data were analysed using a linear mixed‐effect model (*n *= 14). All data are presented as mean bars plus individual participant response lines.

**FIGURE 2:**
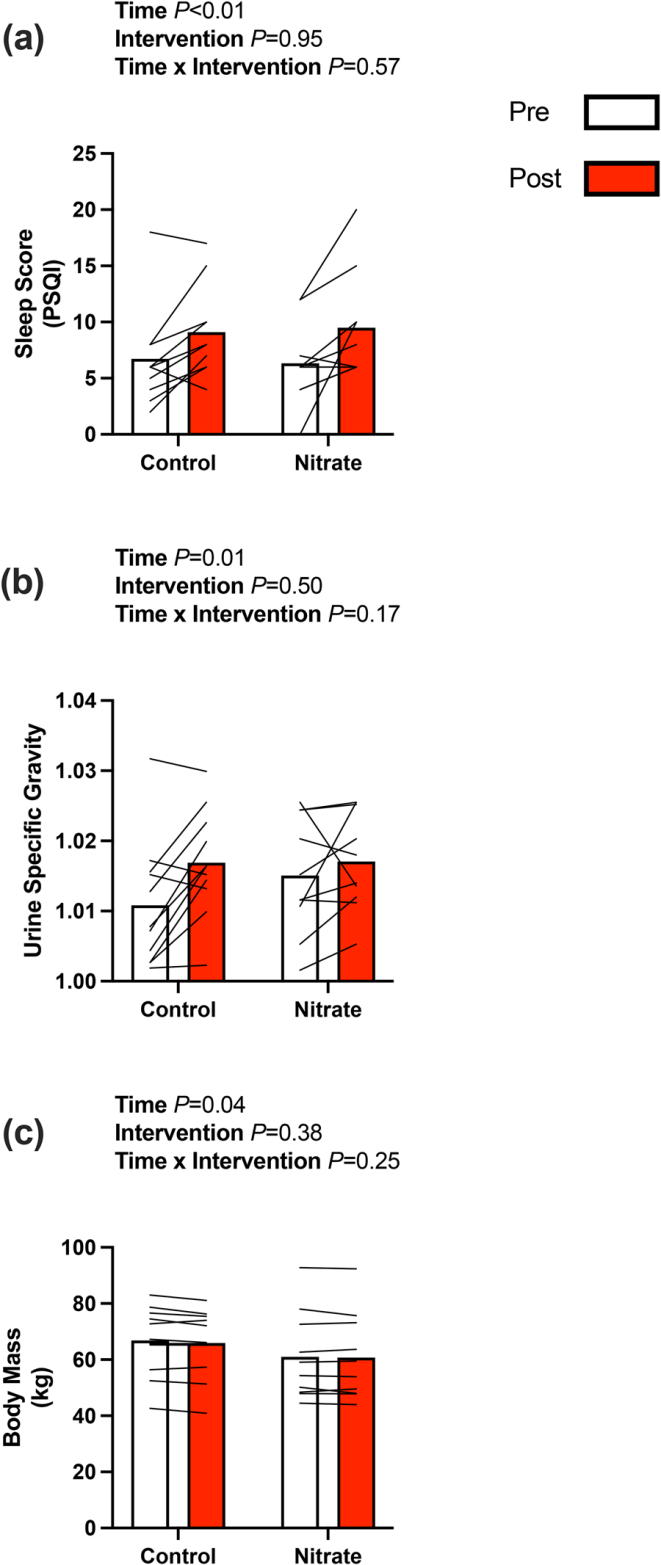
Changes in the Pittsburgh Sleep Quality Index (a), urine specific gravity (b) and body mass (c) following the 10 day Ramadan fasting intervention, with or without dietary nitrate supplementation. Data were analysed using a linear mixed‐effect model (*n *= 21). All data are presented as mean bars plus individual participant response lines.

Table 2 presents the cardiorespiratory responses obtained during the exercise tests. Based on the criteria outlined by Wagner et al. (2020), 37 of 41 participant tests met the criteria for performing a maximal exercise test. Of those four that did not, one occurred during the control pre‐trial, one during the control post‐trial, and two during the dietary nitrate post‐trial. No main effect of time (all *P *> 0.14) or interaction effects (all *P *> 0.16) were detected for peak exercise measurements of ratings of perceived exertion, respiratory exchange ratio, minute ventilation, diastolic blood pressure, or cardiac output. A main effect of time existed for peak systolic blood pressure (*P* = 0.02) and trends existed for peak heart rate (*P* = 0.07), first ventilatory threshold (*P* = 0.08) and respiratory compensation point (*P* = 0.06), all being lower following 10 days of Ramadan fasting. A interaction effect (*P *= 0.048) was noted for the calculated arteriovenous oxygen difference, with values decreasing in control subjects but increasing in the dietary nitrate group [∆, −1.4 ± 2.2 vs. 0.9 ± 2.4 mL O_2_ (100 mL)^−1^, η^2^_p_ = 0.22, *P *= 0.048]. A significant interaction effect was also detected for absolute ${\dot{V}}_{O_{2}\mathrm{peak}}$ (η^2^_p_ = 0.33, *P *< 0.01), relative ${\dot{V}}_{O_{2}\mathrm{peak}}$ (η^2^_p_ = 0.28, *P *= 0.02) and peak power output (η^2^_p_ = 0.22, *P *= 0.04), with each measure demonstrating reductions over time in the control group but not in the dietary nitrate group (Figure 3). The changes in exercise systolic blood pressure per metabolic equivalent were different between groups (∆, 0.78 ± 1.98 vs. −2.08 ± 1.61 mmHg MET^−1^, η^2^_p_ = 0.39, *P *< 0.01). Exploratory analysis of sex did not detect any group × time × sex interactions for any measure (all *P *> 0.65).

**TABLE 1:** Baseline characteristics.

Characteristic	Control (*n *= 11)	Nitrate (*n *= 10)	*P*‐value
Female subjects, *n*	6	7	0.66
Age, years	22 ± 4	19 ± 1	0.02
Height, cm	167 ± 6	163 ± 9	0.33
Body mass, kg	67 ± 12	61 ± 16	0.35
Resting HR, beats min^−1^	78 ± 12	73 ± 9	0.27
Resting SBP, mmHg	112 ± 11	102 ± 11	0.06
Resting DBP, mmHg	75 ± 8	70 ± 6	0.13
${\dot{V}}_{O_{2}\mathrm{peak}}$, mL kg^−1^ min^−1^	34 ± 7	31 ± 8	0.38
Peak power output, W	220 ± 64	181 ± 71	0.20

*Notes*: Values represent the mean ± SD. Data were analysed using unpaired Student's *t*‐tests.

Abbreviations: DBP, diastolic blood pressure; HR, heart rate; SBP, systolic blood pressure; ${\dot{V}}_{O_{2}\mathrm{peak}}$, peak oxygen uptake.

**TABLE 2:** Perceptual and cardiorespiratory measures before and after 10 days of Ramadan fasting in the control and dietary nitrate groups.

Parameter	Control (*n *= 11) Pre Post		Nitrate (*n *= 10) Pre Post		Group *P*‐value	Time *P*‐value	Group × time *P*‐value
RPE	17.8 ± 2.0	17.5 ± 1.7	15.6 ± 3.2	15.7 ± 2.9	0.02	0.86	0.76
RER	1.3 ± 0.1	1.3 ± 0.1	1.3 ± 0.1	1.3 ± 0.1	0.43	0.35	0.46
Peak ${\dot{V}}_{E}$, L min^−1^	101.5 ± 37.5	93.3 ± 24.7	93.3 ± 40.8	84.9 ± 31.9	0.46	0.44	0.99
Peak SBP, mmHg	181 ± 20	176 ± 19	172 ± 35	163 ± 28	0.29	0.04	0.55
Peak DBP, mmHg	82 ± 9	81 ± 9	82 ± 7	83 ± 6	0.69	0.81	0.52
Peak heart rate, beats min^−1^	189 ± 8	186 ± 10	191 ± 7	189 ± 11	0.53	0.07	0.94
Peak $\dot{Q}$, L min^−1^	15.2 ± 2.5	15.5 ± 2.4	14.1 ± 3.3	14.1 ± 5.1	0.40	0.75	0.80
Peak arteriovenous oxygen difference, mL O_2_ (100 mL)^−1^	14.9 ± 4.8	13.5 ± 4.7	13.2 ± 4.0	14.1 ± 2.7	0.78	0.67	0.048
SBP/MET slope, mmHg MET^−1^	8.0 ± 1.8	8.8 ± 2.7	8.7 ± 2.2	6.8 ± 1.1	0.37	0.15	<0.01
VT1, mLmin^−1^	1516.1 ± 357.9	1496.7 ± 401.4	1336.9 ± 534.1	1270.0 ± 493.7	0.36	0.08	0.32
RCP, mL min^−1^	2026.7 ± 536.4	1952.8 ± 607.1	1720.6 ± 714.4	1645.0 ± 731.4	0.34	0.06	0.98

*Notes*: Values are means ± SD. Data were analysed using linear mixed‐effects models.

Abbreviations: DBP, diastolic blood pressure; MET, metabolic equivalents; *Q̇*, cardiac output; RCP, respiratory compensation point; RER, respiratory exchange ratio; RPE, rating of perceived exertion; SBP, systolic blood pressure; ${\dot{V}}_{E}$, ventilation; VT1, first ventilatory threshold.

**FIGURE 3:**
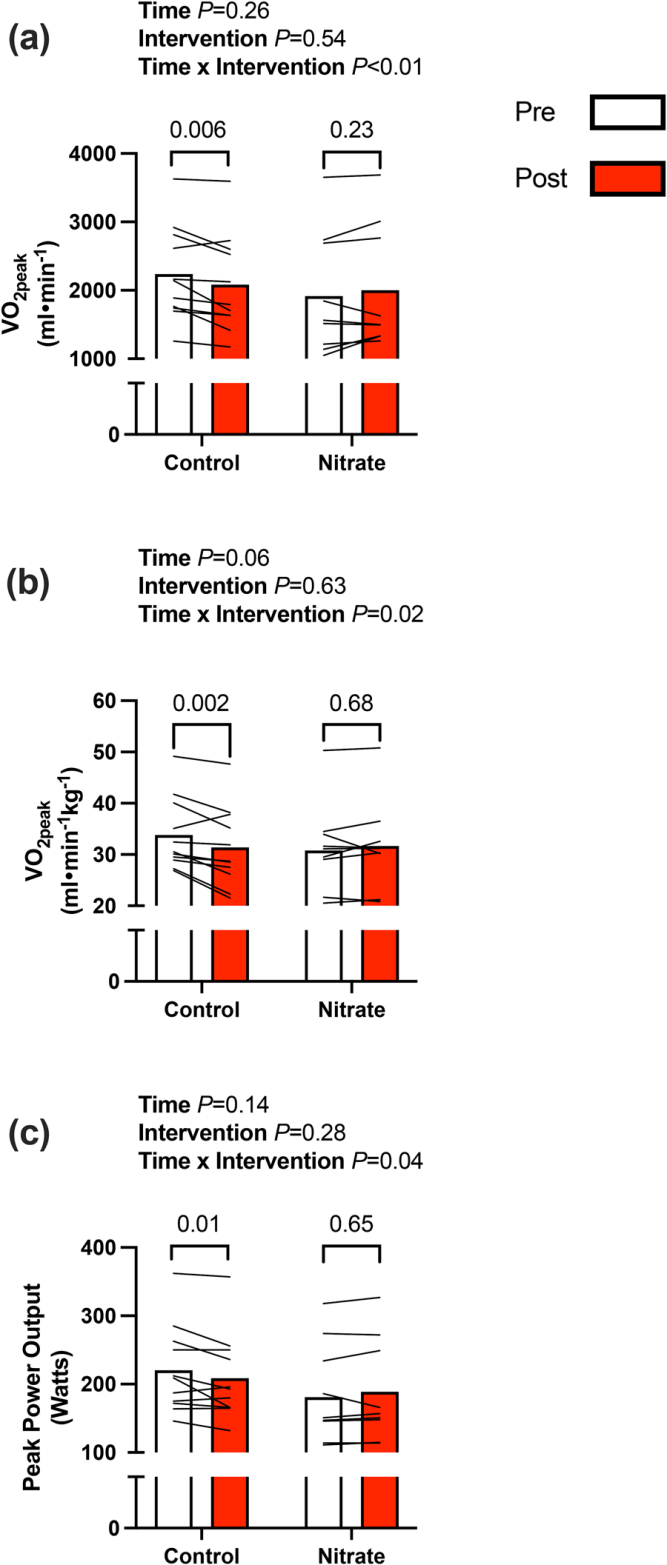
Changes in absolute peak oxygen uptake (${\dot{V}}_{O_{2}\mathrm{peak}}$) (a), relative ${\dot{V}}_{O_{2}\mathrm{peak}}$ (b) and peak power output (c) following the 10 day Ramadan fasting intervention, with or without dietary nitrate supplementation. Data were analysed using a linear mixed‐effect model (*n *= 21). All data are presented as mean bars plus individual participant response lines.

## 4 DISCUSSION

In the present study, we investigated whether daily dietary nitrate supplementation attenuates reductions in cardiorespiratory fitness during 10 days of Ramadan fasting. In line with our hypothesis, beetroot juice supplementation mitigated the decline in ${\dot{V}}_{O_{2}\mathrm{peak}}$ and peak power output observed in the control group. In contrast, changes in body mass and estimated peak cardiac output did not differ between groups. Across all participants, Ramadan fasting was associated with reductions in sleep quality, impaired urinary hydration status and decreased frequency of habitual physical activity. Collectively, these findings suggest that dietary nitrate supplementation attenuates Ramadan‐associated reductions in ${\dot{V}}_{O_{2}\mathrm{peak}}$, potentially via peripheral mechanisms influencing oxygen extraction and skeletal muscle function.

### 4.1 Ramadan and ${\dot{V}}_{O_{2}\mathrm{peak}}$

Several prior studies have examined the effects of Ramadan fasting on cardiorespiratory fitness and exercise performance. In the largest trial to date, Roy and Bandyopadhyay (2015) examined 77 untrained Muslim males, showing a progressive decline in relative ${\dot{V}}_{O_{2}\mathrm{peak}}$ during each week of Ramadan, which recovered after returning to an uncontrolled dietary pattern. Sweileh et al. (1992) reported a decline in ${\dot{V}}_{O_{2}\mathrm{peak}}$ among six healthy Muslims during the first week of Ramadan that recovered by the fourth week of Ramadan, aligning with changes in hydration status. Finally, a study in 18 well‐trained middle‐distance runners reported no change in ${\dot{V}}_{O_{2}\mathrm{peak}}$ but an increase in the time constant of oxygen kinetics and reduced running performance during the last week of Ramadan (Brisswalter et al., 2011). The present results add to the literature demonstrating a ∼6% reduction in ${\dot{V}}_{O_{2}\mathrm{peak}}$ and a ∼5% drop in peak power output in the control group after 10 days of Ramadan.

The potential mechanisms responsible for a reduction in ${\dot{V}}_{O_{2}\mathrm{peak}}$ during Ramadan fasting are likely to be multifactorial and have been hypothesized to include mild dehydration, decreases in muscle glycogen storage, sudden decreases in physical activity causing skeletal muscle atrophy, and poor sleep quality inducing central fatigue and reducing neural drive to the exercising muscles (Bouhlel et al., 2013; Farooq et al., 2021; Jandali et al., 2024; Sweileh et al., 1992; Tornero‐Aguilera et al., 2022). Reduced physical activity has been associated with declines in oxidative enzyme activity, capillary density and muscle perfusion (Bengt & Gollnick, 1983; Saltin et al., 1968), all of which can reduce skeletal muscle oxygen extraction (McGuire et al., 2001). Even modest changes in daily step count can have implications for ${\dot{V}}_{O_{2}\mathrm{peak}}$ (Cao et al., 2009; Pillay et al., 2014). Moreover, prior work has reported reductions in fat‐free mass during Ramadan (Fernando et al., 2019; Najafi et al., 2023), which might further compromise oxygen utilization and exercise performance.

### 4.2 Dietary nitrate supplementation and ${\dot{V}}_{O_{2}\mathrm{peak}}$

The preservation of ${\dot{V}}_{O_{2}\mathrm{peak}}$ in the dietary nitrate supplementation group without corresponding group differences in estimated peak cardiac output suggest that dietary nitrate might act through a peripheral mechanism. In support, we observed a trend towards a greater calculated arteriovenous oxygen difference in the dietary nitrate supplementation group. Dietary nitrate has been shown to enhance skeletal muscle perfusion and mitochondrial efficiency, reduce the oxygen cost of exercise and enhance contractile efficiency (Bailey et al., 2015; Gao et al., 2021; Larsen et al., 2011). These peripheral effects might be particularly relevant in conditions of reduced habitual physical activity and daily fluid intake during Ramadan.

Although we did not directly measure muscle mass or mitochondrial function, these findings align with the possibility that nitrate supplementation preserved peripheral determinants of ${\dot{V}}_{O_{2}\mathrm{peak}}$, consistent with the observed differences in cardiorespiratory fitness. Prior animal and human studies support a protective role of nitrate during periods of reduced physical activity. Nazari et al. (2024) reported that dietary nitrate supplementation with beetroot extract could attenuate the decline in gastrocnemius muscle cross‐sectional area and weight in mice undergoing 7 days of hindlimb immobilization. Petrick et al. (2023) reported that dietary nitrate could prevent declines in mitochondrial protein synthesis rates, markers of mitochondrial content and mitochondrial respiration following immobilization. Future studies are required to elucidate the mechanisms by which dietary nitrate prevents a decline in ${\dot{V}}_{O_{2}\mathrm{peak}}$ during Ramadan.

### 4.3 Exercise blood pressure

Ramadan fasting has been shown to reduce both systolic and diastolic blood pressure at rest independent of changes in body weight and total body water (Al‐Jafar et al., 2021). Dietary nitrate has been shown to lower resting and exercise blood pressure (Jones, 2014). In the present study, peak systolic blood pressure did not differ between groups, but the rise in systolic blood pressure relative to metabolic demand was attenuated in the dietary nitrate group owing to the changes in ${\dot{V}}_{O_{2}\mathrm{peak}}$. Prior work has shown in middle‐aged cohorts that systolic blood pressure per metabolic equivalent responses to exercise are independently associated with future all‐cause mortality (Hedman et al., 2020). Whether dietary nitrate can be used to manage this risk factor in at‐risk populations requires warrants future study.

### 4.4 Methodological considerations

We acknowledge several limitations in the present study. First, the absence of a placebo control introduces the possibility of expectancy effects, although we did not detail the study hypothesis to participants, and ratings of perceived exertion and the respiratory exchange ratio were similar between groups. Second, lean body mass was not assessed directly, limiting interpretation of compositional changes that could alter ${\dot{V}}_{O_{2}\mathrm{peak}}$. Third, physical activity was assessed via questionnaire rather than objective measures, such as actigraphy. Fourth, although plasma NO_x_ concentrations confirmed effective nitrate supplementation, the study design cannot fully distinguish acute from cumulative effects. However, given that the average half‐life of plasma nitrate is 5 h (Arazi & Eghbali, 2021) and that only three participants were tested within this window, the acute effects of the final dose were likely to be minor in comparison to the cumulative effects of daily supplementation. Future research with longer washout periods is required to distinguish the acute versus chronic effects of dietary nitrate supplementation. Finally, the study involved recreationally active adults, and extrapolation to elite athletes or alternative populations warrants replication.

### 4.5 Perspectives

Ramadan fasting differs from non‐religious time‐restricted feeding protocols owing to prolonged daytime fluid restriction, altered sleep schedules and reduced habitual physical activity. Although non‐religious intermittent fasting might preserve or improve ${\dot{V}}_{O_{2}\mathrm{peak}}$ (Correia et al., 2020), Ramadan might impair cardiorespiratory fitness. Given that reduced cardiorespiratory fitness is linked to diminished exercise tolerance and adverse health outcomes (Ross et al., 2016), strategies to preserve ${\dot{V}}_{O_{2}\mathrm{peak}}$ might be relevant for physically active individuals during Ramadan. Dietary nitrate is already recognized as a potential ergogenic aid (Maughan et al., 2018), providing a strong background rationale for its use in situations of reduced physical activity. This can be important for Muslim athletes, who can demonstrate impaired performance during Ramadan (Brisswalter et al., 2011; Lipert et al., 2021; Trabelsi et al., 2024). Future studies should consider the duration of fasting during Ramadan, which can differ based on geographical location and the calendar season, in addition to determining whether preservation of ${\dot{V}}_{O_{2}\mathrm{peak}}$ translates to sport‐specific performance.

## 5 CONCLUSION

Daily dietary nitrate supplementation using beetroot juice attenuated the decline in ${\dot{V}}_{O_{2}\mathrm{peak}}$ and peak power output during 10 days of Ramadan fasting in healthy young adults. These findings support a predominantly peripheral mechanism, likely to involve enhanced skeletal muscle oxygen extraction, and suggest that nitrate supplementation might help to maintain aerobic capacity in prolonged daytime fasting conditions.

## AUTHOR CONTRIBUTIONS

Mikail M. Bhimani, Julian C. Bommarito and Philip J. Millar conceived and designed research. Mikail M. Bhimani, Julian C. Bommarito, Natalie I. Miners, Pardeep K. Khangura, Rileigh K. Stapleton, Ashly Sharma, Johan S. Thiessen, Nicolas O. Cyr, Devin G. McCarthy and Jianzhang Dong performed experiments. Mikail M. Bhimani and Julian C. Bommarito analysed data. Clara E. Cho assisted with metabolite quantification. Mikail M. Bhimani, Julian C. Bommarito and Philip J. Millar interpreted the results of experiments. Mikail M. Bhimani prepared figures and drafted the manuscript. Mikail M. Bhimani, Devin G. McCarthy, Clara E. Cho and Philip J. Millar revised the manuscript. All authors approved the final version of manuscript and agree to be accountable for all aspects of the work in ensuring that questions related to the accuracy or integrity of any part of the work are appropriately investigated and resolved. All persons designated as authors qualify for authorship, and all those who qualify for authorship are listed.

## CONFLICT OF INTEREST

None declared.

## GENERATIVE AI STATEMENT

The authors confirm that no artificial intelligence tools, including large language models (LLMs), were used in the drafting or revision of this manuscript. All content was conceived, written and approved solely by the authors.

## Data Availability

This data are available upon reasonable request from the corresponding author.
